# Improving farm productivity and profitability through AI diffusion: evidence from Punjab, Pakistan

**DOI:** 10.3389/frai.2026.1890862

**Published:** 2026-10-21

**Authors:** Aeman Khalid, Colin Akhurst, Sayed Abdul Majid Gilani, Firas Armosh, Syeda Aqsa Zanib, Jack Boath, Richard Stanley Peel, Yohani Jinadasa

**Affiliations:** 1 Ulster Business School, Ulster University, Birmingham, United Kingdom; 2 Birmingham City University Business School, Birmingham City University, Birmingham, United Kingdom; 3 Coventry Business School, Coventry University, Astana, Kazakhstan; 4 Bath Spa Business School, Bath Spa University, Bath, United Kingdom; 5 Operations Department, Emirates Specializing Contracting and Oilfield Services (ESCO) LLC, Abu Dhabi, United Arab Emirates; 6 The School of Education, University of Kurdistan Hewler, Erbil, Iraq; 7 Westford Business School, Westford University College, Sharjah, United Arab Emirates

**Keywords:** agriculture, artificial intelligence, farm productivity, innovation diffusion, Pakistan

## Abstract

**Introduction:**

Agriculture remains central to Pakistan's economy, with Punjab accounting for the largest share of national crop production. Despite the rapid global advancement of artificial intelligence (AI)-enabled agricultural technologies, their diffusion across farms in developing economies remains uneven and underexplored. This study examines the extent of AI diffusion among farmers in Punjab, Pakistan, and assesses its associations with perceived farm productivity and profitability.

**Methods:**

Drawing on Diffusion of Innovations (DoI) theory, complemented by the Resource-Based View (RBV) and the Technology-Organisation-Environment (TOE) framework, the study adopts a quantitative, cross-sectional design using survey data from 101 farmers.

**Results:**

The findings indicate that while awareness and perceived benefits of AI are relatively high, actual adoption remains moderate (AI Diffusion: *M* = 18.27, SD = 3.71), indicating a gap between perceived value and implementation. AI diffusion was positively correlated with perceived farm productivity (*r* = 0.617, *p* < 0.001) and perceived farm profitability (*r* = 0.607, *p* < 0.001). The findings therefore demonstrate statistically significant associations between greater AI diffusion and stronger perceived farm performance, while not establishing causal effects.

**Discussion:**

The observed perception-adoption gap is consistent with structural barriers identified in previous research, including high investment costs, limited digital infrastructure and technical capability constraints. The study contributes context-specific evidence on digital agriculture in a developing economy by jointly examining AI diffusion, productivity and profitability. The findings highlight the importance of infrastructure, institutional support and capability development in facilitating wider and more effective adoption of AI-enabled agricultural technologies.

## 1 Introduction

Agriculture remains a cornerstone of economic development, employment and food security across many developing economies. In Pakistan, the agricultural sector contributes substantially to national gross domestic product and rural livelihoods, with Punjab accounting for the largest share of crop production, including wheat, rice, cotton and sugarcane (Abbas et al., 2022). Despite its strategic importance, agricultural productivity remains constrained by inefficient resource utilization, climate variability, increasing production costs and persistent structural limitations that reduce farm profitability and long-term sustainability (Nasreen and Ashraf, 2020). These challenges have intensified interest in digital technologies capable of improving decision-making and resource efficiency.

Artificial intelligence (AI) has emerged as one of the most influential digital technologies driving agricultural transformation. Through machine learning, predictive analytics, remote sensing, robotics and precision farming applications, AI supports improved crop monitoring, irrigation management, disease detection, yield forecasting and operational planning (Karunathilake et al., 2023; Kreutzer, 2024; Malgi et al., 2026). Evidence from developed agricultural systems consistently demonstrates that AI-enabled technologies can improve productivity, optimize input utilization and enhance operational efficiency (Araújo et al., 2021). Consequently, AI is increasingly recognized as a key component of Agriculture 4.0 [for example, Deere & Company (John Deere): Moline, Illinois, USA] and sustainable agricultural development.

By contrast, the diffusion of AI remains highly uneven across geographical and economic contexts. While adoption has accelerated within technologically advanced and capital-intensive farming systems, implementation across developing economies remains comparatively limited (Cavazza et al., 2023). In Pakistan, particularly within Punjab where agriculture is dominated by smallholder and resource-constrained farming systems, AI adoption remains at an early stage despite increasing awareness of its potential benefits (Williams-Onyeji, 2025). Existing studies attribute this slow diffusion to multiple structural barriers, including limited digital infrastructure, high investment costs, unreliable energy supply, insufficient technical expertise and constrained institutional support (Elbasi et al., 2022; Gilani and Faccia, 2021).

Although previous research has examined digital agriculture and technology adoption, the empirical literature remains fragmented in three important respects. First, most studies have concentrated on technology adoption or productivity outcomes, while comparatively little attention has been given to farm profitability as a distinct economic outcome (Sood et al., 2022; Tzachor et al., 2022). Second, evidence from developing agricultural economies, particularly Pakistan, remains relatively limited compared with studies undertaken in developed countries. Third, existing research has rarely integrated technological, organizational and environmental perspectives to explain why AI adoption varies across farming systems and how these differences influence economic performance. Consequently, there remains limited empirical understanding of whether AI diffusion is associated with improvements in both farm productivity and profitability within the institutional and structural conditions characterizing Punjab's agricultural sector.

In this study, AI diffusion refers to the extent to which artificial intelligence-enabled agricultural technologies have been adopted and integrated into routine farming activities. Farm productivity is conceptualized as perceived improvements in production efficiency, crop yield and resource utilization resulting from farming practices, while farm profitability refers to farmers' perceived improvements in financial performance, including income generation, cost efficiency and overall economic returns. The operationalisation and measurement of these constructs are described in detail in Section 3.

Empirical research on AI in agriculture has predominantly focused on developed economies, where advanced infrastructure and capital-intensive farming systems facilitate rapid adoption. Studies consistently report positive impacts on productivity, efficiency, and environmental sustainability (Araújo et al., 2021). These findings are not easily generalisable to developing-country contexts.

In emerging economies, evidence remains fragmented and context-dependent. While some studies demonstrate the potential of digital advisory services and AI-enabled tools to improve productivity, adoption rates remain relatively low due to cost barriers, trust issues, and capability constraints. In Pakistan, existing research has largely focused on ICT adoption and productivity outcomes, with limited attention to profitability and long-term economic sustainability (Sood et al., 2022).

A key limitation of the existing literature is the overemphasis on productivity as the primary outcome variable, often at the expense of profitability. Given that farmers' adoption decisions are ultimately driven by economic returns, this represents a significant conceptual and empirical gap. Furthermore, there is limited research examining how structural constraints mediate the relationship between AI adoption and economic performance.

Another important gap relates to the limited theoretical integration within the existing literature. Many studies have examined farmer technology adoption through individual theoretical perspectives, despite evidence that no single theory fully captures the multidimensional nature of technology adoption in complex agricultural environments (Karbo et al., 2024). As recent research suggests, the benefits of AI are contingent upon the interaction between technological capabilities, organizational resources, and environmental conditions (Tzachor et al., 2022; Gardezi et al., 2024).

To address these gaps, this study examines AI diffusion among farmers in Punjab, Pakistan, and investigates its relationship with farm productivity and profitability. The research is theoretically informed by Diffusion of Innovations (DoI) theory and complemented by the Resource-Based View (RBV) and the Technology–Organization–Environment (TOE) framework to provide a multi-level explanation of technology adoption and farm performance. Using quantitative survey data collected from farmers in Punjab, the study examines AI adoption patterns, perceived economic outcomes and the structural conditions influencing technology diffusion.

The study contributes to the literature in three ways. First, it provides context-specific empirical evidence from a developing agricultural economy where AI adoption remains comparatively underexplored, helping to address the geographical imbalance in the existing literature. Second, it advances understanding of farm-level economic performance by jointly examining productivity and profitability rather than treating productivity as the sole indicator of technological success. Third, it integrates the DoI, RBV and TOE frameworks to provide a multi-level theoretical explanation of how technological, organizational and environmental factors jointly influence AI diffusion and associated economic outcomes. Collectively, the findings contribute to the growing literature on digital agricultural transformation while providing practical insights for policymakers, agricultural practitioners and researchers seeking to promote more inclusive and sustainable AI adoption within emerging agricultural economies.

## 2 Literature review

### 2.1 Artificial intelligence technologies and digital transformation in agriculture

Artificial intelligence in agriculture encompasses a diverse range of technologies rather than a single innovation. These include machine learning, deep learning, computer vision, robotics, Internet of Things (IoT), big data, cloud computing, and intelligent decision-support systems, each supporting different aspects of agricultural production and management (Gilani et al., 2023). Predictive AI supports yield forecasting, weather prediction and pest outbreak modeling through machine learning algorithms. Descriptive AI processes and visualizes agricultural data generated from sensors, satellite imagery and Internet of Things (IoT) devices to improve situational awareness. Analytical AI analyses large agricultural datasets to optimize resource allocation, fertilizer application, and irrigation scheduling, while cognitive AI supports farm decision-making through intelligent recommendation systems and autonomous technologies such as agricultural robotics and precision machinery (Wakchaure et al., 2023). Collectively, these technologies form the technological foundation of Agriculture 4.0, supporting activities across the agricultural value chain, including crop planning, production, resource management, harvesting and market decision-making. Through these applications, AI enables more efficient, data-driven farm management and has the potential to improve both operational performance and economic outcomes.

Artificial intelligence (AI) is increasingly recognized as a general-purpose technology (GPT) with the capacity to transform economic systems through widespread productivity enhancements and structural change. In agriculture, AI operates at the intersection of multiple digital technologies, including machine learning, remote sensing, Internet of Things (IoT), big data analytics, and robotics, collectively forming the foundation of what is often termed Agriculture 4.0 (Kreutzer, 2024; Inoue, 2020; Gilani et al., 2025a).

Unlike earlier waves of agricultural innovation, including mechanization and the Green Revolution, AI enables continuous, data-driven optimisation of farming practices, shifting the sector from reactive decision-making to predictive and prescriptive systems. This transition supports more efficient resource allocation, improved yield forecasting, and enhanced resilience to environmental uncertainty (Gilani et al., 2023; Karunathilake et al., 2023; Rai et al., 2023). For instance, AI-powered disease detection systems and precision irrigation technologies have been shown to reduce input waste while simultaneously improving crop health and productivity outcomes.

Empirical evidence suggests that AI adoption can generate substantial productivity gains, with some studies estimating improvements in the range of 10%−30% depending on context and level of technological integration (Araújo et al., 2021; Gilani et al., 2024; Krupicka et al., 2025). Such estimates are highly contingent upon complementary factors, including data quality, infrastructure, and technical expertise. This has led to the recognition that AI's benefits are not inherent to the technology itself but are co-produced through socio-technical systems, where human capabilities, institutional environments, and technological tools interact (Elbasi et al., 2022).

Recent scholarship has begun to challenge the predominantly techno-optimistic narrative surrounding AI in agriculture (Cavazza et al., 2023; Songol et al., 2024; Aderibigbe et al., 2023). Critics argue that AI adoption may exacerbate structural inequalities, particularly in developing economies where access to digital infrastructure, capital, and knowledge is uneven (Cavazza et al., 2023). Large-scale farms and agribusinesses are better positioned to invest in AI technologies, while smallholder farmers face significant barriers, including affordability constraints, limited digital literacy, and a lack of institutional support.

AI-driven transformation in agriculture is increasingly viewed not as a uniform process but as a differentiated and uneven phenomenon, shaped by context-specific socio-economic, organizational and institutional conditions (Cavazza et al., 2023; Songol et al., 2024; Aderibigbe et al., 2023). This necessitates a shift from viewing AI purely as a technological innovation to understanding it as part of a broader digital ecosystem, where adoption and impact depend on the alignment of multiple enabling factors.

While these technological advances demonstrate the transformative potential of AI, understanding their value requires distinguishing between improvements in technical efficiency and broader economic performance. The following section therefore examines the conceptual relationship between farm productivity and profitability.

### 2.2 Productivity and profitability: conceptual distinctions and linkages

Productivity and profitability represent two central yet analytically distinct dimensions of agricultural performance. Productivity is typically defined in terms of technical efficiency, often measured through Total Factor Productivity (TFP), which captures the ability of producers to convert inputs into outputs (Villoria, 2019). In contrast, profitability reflects economic efficiency, accounting for revenues, costs, and market dynamics (Foster and Rosenzweig, 2022).

While the literature has traditionally treated productivity improvements as a proxy for economic success, more recent empirical research suggests that the relationship between productivity and farm profitability is more complex than previously assumed, with profitability influenced not only by productivity gains but also by relative prices, production costs, and broader market conditions (Mugera et al., 2016). Specifically, increases in productivity do not necessarily lead to corresponding gains in profitability because economic outcomes are also shaped by adoption costs, relative prices, market volatility, and institutional constraints (Mugera et al., 2016; Foster and Rosenzweig, 2022). This divergence has been conceptualized as the productivity–profitability paradox (Benton and Bailey, 2019), highlighting the importance of evaluating technological innovations in terms of both technical efficiency and broader economic performance (Tzachor et al., 2022).

From a microeconomic perspective, farmers' adoption decisions are guided by expected marginal returns under uncertainty. AI technologies can enhance technical efficiency by improving decision-making and resource allocation; however, their economic value depends on whether these efficiency gains outweigh the costs associated with acquisition, implementation, and maintenance (Gilani et al., 2023). In early adoption stages, high upfront costs and learning requirements may suppress profitability, even where productivity improvements are evident.

Furthermore, the relationship between productivity and profitability is mediated by external factors, including input prices, output markets, and policy environments. For example, improved yields may not translate into higher profits if market prices decline or if increased production leads to oversupply. Conversely, cost reductions achieved through AI-enabled efficiency gains may enhance profitability even in the absence of significant output expansion.

Recent research increasingly emphasizes the importance of adopting a dynamic perspective (Tzachor et al., 2022), recognizing that the economic benefits of technology adoption may evolve over time. While short-term profitability may be constrained, long-term gains can emerge through cumulative learning effects, economies of scale, and improved capability development (Tzachor et al., 2022).

This literature highlights the need for an integrated analytical framework that jointly examines productivity and profitability, rather than treating them as independent outcomes. Such an approach is particularly important in developing-country contexts, where resource constraints and risk factors are more pronounced.

Although productivity and profitability provide important indicators of farm performance, their relationship is strongly influenced by the institutional and structural conditions within which technologies are adopted. These contextual factors are particularly relevant in Punjab, Pakistan, and are discussed in the following section.

### 2.3 Agricultural context of Punjab: structural constraints and opportunities

Punjab is widely recognized as the agricultural backbone of Pakistan, contributing a significant proportion of the country's crop production and rural employment (Raza et al., 2019). Despite its strategic importance, the region faces persistent structural constraints that limit productivity growth and technological advancement.

Environmental challenges, including water scarcity, soil degradation, and climate variability, have intensified in recent years, placing increasing pressure on agricultural systems (Nasreen and Ashraf, 2020). Over-reliance on irrigation has led to declining groundwater levels, while climate change has increased the frequency of extreme weather events, such as droughts and floods (Gilani et al., 2023). These factors contribute to yield instability and increased production risk.

In addition to environmental pressures, institutional and infrastructural limitations play a critical role in shaping agricultural performance. Limited broadband connectivity, unreliable electricity supply, and underdeveloped extension services restrict farmers' access to digital technologies and information (Gilani et al., 2023). The predominance of smallholder farming further complicates adoption dynamics, as smaller farms often lack the financial resources and technical capabilities required to invest in advanced technologies (Kousar et al., 2020).

Recent policy initiatives have sought to promote digital transformation within Pakistan's agricultural sector through investments in digital infrastructure, precision agriculture and technology extension programmes (Nadeem et al., 2024). However, the implementation and impact of these initiatives have remained uneven across regions and farm types. Technologically advanced commercial farms increasingly coexist alongside smallholder farms that continue to rely on conventional agricultural practices. This uneven diffusion of digital technologies raises concerns regarding widening productivity disparities, unequal access to innovation, and the long-term competitiveness of resource-constrained farming systems as digital agriculture becomes increasingly integral to sustainable agricultural development.

Within this context, AI adoption represents both an opportunity and a challenge. While AI has the potential to enhance productivity, reduce risk, and improve sustainability, its successful diffusion depends on overcoming deeply embedded structural constraints. Understanding these contextual dynamics is therefore essential for analyzing the adoption and impact of AI in Punjab.

These contextual characteristics demonstrate that technology adoption cannot be explained solely by technical considerations. A broader theoretical perspective is therefore required to account for behavioral, organizational and environmental influences on AI diffusion.

### 2.4 Theoretical integration: a multi-level perspective on AI diffusion

To capture the complexity of AI adoption in agriculture, this study adopts a multi-theoretical framework integrating Diffusion of Innovations (DoI), the Resource-Based View (RBV), and the Technology–Organization–Environment (TOE) framework.

Diffusion of Innovations (DoI) theory provides a foundational perspective on how new technologies spread within a social system. According to Rogers, adoption decisions are influenced by five perceived characteristics of an innovation: relative advantage (the perceived benefits over existing practices), compatibility (consistency with existing values, needs and farming practices), complexity (the perceived difficulty of understanding and using the innovation), trialability (the opportunity to experiment before full adoption) and observability (the visibility of innovation outcomes to others) (Assidi et al., 2025). Within the context of AI-enabled agriculture, relative advantage reflects farmers' perceptions that digital technologies can improve production efficiency, optimize resource utilization, enhance decision-making and strengthen overall farm performance. These operational improvements may subsequently contribute to improved productivity and profitability. While DoI is widely applied in agricultural research, it has been criticized for emphasizing innovation characteristics and individual adoption decisions while giving comparatively less attention to broader structural, institutional, and contextual constraints that shape technology adoption (Karbo et al., 2024). These limitations are particularly evident in developing agricultural economies, where infrastructure, policy support, institutional capacity, and resource availability play a significant role in shaping farmers' adoption decisions (Smidt and Jokonya, 2023; Karbo et al., 2024).

The Resource-Based View (RBV) addresses this limitation by focusing on the role of internal capabilities and resources in shaping adoption outcomes. According to RBV, competitive advantage arises from resources that are valuable, rare, and difficult to imitate, such as technical knowledge, financial capital, and organizational capabilities (Helfat et al., 2023). In the context of AI, RBV suggests that adoption alone is insufficient; rather, the ability to effectively integrate and utilize AI technologies depends on the presence of complementary capabilities.

The Technology–Organization–Environment (TOE) framework complements these perspectives by incorporating external environmental factors, including institutional support, regulatory conditions, and market dynamics (Gilani et al., 2025b). This is particularly relevant in developing economies, where infrastructural deficits and policy environments play a decisive role in shaping adoption behavior.

The integration of DoI, RBV and TOE provides a complementary theoretical perspective capable of explaining AI diffusion across multiple analytical levels. DoI explains farmers' behavioral intentions to adopt AI technologies based on perceived innovation characteristics. RBV explains how differences in internal resources and organizational capabilities influence successful implementation and value creation. TOE extends the analysis by recognizing that technological readiness, institutional support and environmental conditions shape adoption opportunities and constraints. Taken together, these perspectives provide a comprehensive theoretical explanation of AI diffusion and the factors associated with differences in farm productivity and profitability. Rather than attributing outcomes to technology alone, the integrated framework recognizes that organizational capabilities, resource availability and external institutional conditions shape both AI adoption and its potential economic benefits.

### 2.5 Integrated theoretical framework

Building upon the preceding theoretical discussion, this study integrates the Diffusion of Innovations (DoI) theory, the Resource-Based View (RBV) and the Technology–Organization–Environment (TOE) framework to develop a conceptual model explaining AI diffusion and its relationship with farm productivity and profitability within the institutional context of Punjab, Pakistan. The integration of these complementary perspectives enables adoption to be examined from individual, organizational and environmental viewpoints, providing a more comprehensive explanation than any single theory alone.

Figure 1 presents the integrated theoretical framework underpinning this study. It synthesizes the complementary roles of Diffusion of Innovations (DoI), the Resource-Based View (RBV) and the Technology–Organization–Environment (TOE) framework in explaining AI diffusion within the agribusiness context. Specifically, DoI provides the behavioral perspective by explaining how farmers' perceptions of innovation characteristics influence adoption decisions. RBV highlights the importance of internal organizational resources and capabilities for the successful implementation and value creation of AI technologies, while TOE emphasizes the influence of external environmental conditions, including institutional support, digital infrastructure and policy environments.

**Figure 1:**
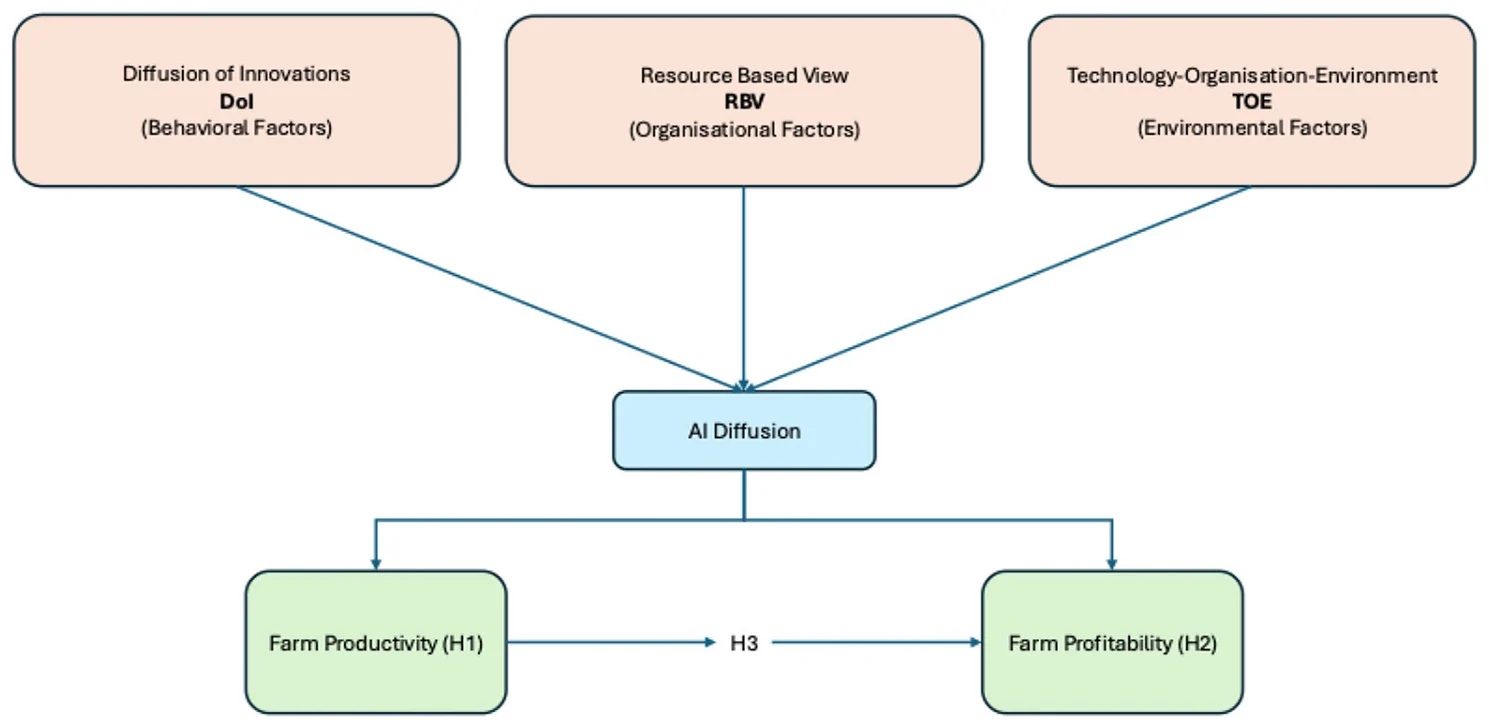
Integrated conceptual framework illustrating the relationship between the theoretical foundations, AI diffusion, and farm performance outcomes. Developed by the authors based on Everett M. Rogers (Diffusion of Innovations), Jay Barney (Resource-Based View), and Louis G. Tornatzky and Mitchell Fleischner (Technology–Organization–Environment Framework).

Collectively, these theoretical perspectives provide a comprehensive explanation of AI diffusion by recognizing that technology adoption is shaped simultaneously by behavioral, organizational and contextual factors. Building on this integrated framework, the study advances the following hypotheses:

**H1:** AI diffusion is positively associated with farm productivity.
**H2:** AI diffusion is positively associated with farm profitability.
**H3:** Farm productivity is positively associated with farm profitability.

These hypotheses reflect the expectation that greater diffusion of AI-enabled technologies will be positively associated with perceived farm productivity and profitability, and that perceived farm productivity will be positively associated with perceived farm profitability. The integrated framework therefore serves as the theoretical foundation for the study's conceptual model and empirical analysis.

## 3 Methodology

### 3.1 Study area and research design

The study was conducted in Punjab, Pakistan's principal agricultural province and the country's largest contributor to the production of major crops, including wheat, rice, cotton and sugarcane. Agriculture represents an important source of rural employment and livelihoods across the province, which is characterized predominantly by smallholder and resource-constrained farming systems alongside a smaller number of larger commercial operations. Despite its agricultural importance, farmers in Punjab face structural constraints relevant to digital technology adoption, including uneven digital connectivity, unreliable electricity supply, limited access to formal credit and gaps in agricultural extension support. These characteristics make Punjab an appropriate setting for examining AI diffusion and its relationship with perceived farm productivity and profitability.

The study adopted a quantitative, cross-sectional survey design. This design was appropriate because the research sought to quantify the extent of AI diffusion and examine statistical associations between AI diffusion, perceived farm productivity and perceived farm profitability at a defined point in time. The study was theoretically informed by Diffusion of Innovations (DoI), the Resource-Based View (RBV) and the Technology–Organization–Environment (TOE) framework. As the design is cross-sectional, the analyses identify associations between the study constructs rather than causal effects.

The research is theoretically informed by Diffusion of Innovations (DoI) theory, complemented by the Resource-Based View (RBV) and the Technology–Organization–Environment (TOE) framework. These perspectives provide the conceptual basis for examining how behavioral perceptions, organizational capabilities and environmental conditions jointly influence AI diffusion and its relationship with productivity and profitability. Given the study's objective of examining associations rather than establishing strict causality, an explanatory cross-sectional design provides an appropriate methodological framework for testing the proposed relationships.

### 3.2 Conceptual model and variable operationalisation

The conceptual model positions AI diffusion as the primary independent variable, with farm productivity and farm profitability as the dependent variables. Productivity is also examined as a predictor of farm profitability, consistent with the theoretical expectation that efficiency gains may contribute to improved financial performance.

AI diffusion was operationalised as a composite perceptual measure capturing farmers' awareness of AI-enabled tools, the extent of their current use, and the degree to which such tools are integrated into routine farming activities (for example, precision farming applications, predictive analytics for yield or pest management, and sensor-based irrigation or monitoring systems). Farm productivity was measured through farmers' perceptions of improvements in production efficiency, crop yield and resource utilization attributable to their farming practices. Farm profitability was measured through perceived improvements in cost efficiency, income stability and overall financial returns.

All constructs were measured using multi-item five-point Likert scales ranging from 1 (strongly disagree) to 5 (strongly agree). The items were adapted from established scales in the technology adoption and digital agriculture literature and were aligned with the theoretical dimensions of the DoI, RBV and TOE frameworks. Perceptual measures were employed because objective farm-level performance data (for example, audited yield or profit figures) are rarely available for small and medium-sized farms in the study context. Such perceptual indicators are widely used in innovation adoption research and capture decision-relevant managerial assessments of technology impact.

Although the instrument included items reflecting farmers' awareness of AI technologies and their intention to expand future AI use, these items formed part of the broader AI diffusion construct and were not operationalised as independent multi-item measures of farmer knowledge or willingness to adopt. Accordingly, they were not introduced retrospectively as separate predictors. Future research should operationalise these dimensions as distinct validated constructs to examine their independent and potentially moderating effects on AI adoption and farm performance.

Table 1 summarizes the operationalisation, measurement and expected direction of the study variables together with the principal literature informing each construct.

**Table 1:** Variable definitions.

Variable	Operationalisation	Measurement	Expected sign	Supporting literature
AI diffusion	Awareness, extent of use, and perceived integration of AI-enabled agricultural tools (e.g., precision farming, predictive analytics, sensor-based systems)	Multi-item 5-point Likert scale (composite score)	+ (H1, H2)	Araújo et al., 2021; Karunathilake et al., 2023
Farm productivity	Perceived improvements in production efficiency, crop yield and resource utilization	Multi-item 5-point Likert scale	+ (H1, H3)	Villoria, 2019; Foster and Rosenzweig, 2022
Farm profitability	Perceived improvements in cost efficiency, income stability and overall financial returns	Multi-item 5-point Likert scale	+ (H2, H3)	Benton and Bailey, 2019; Tzachor et al., 2022

All constructs were measured using multi-item five-point Likert scales (1 = strongly disagree to 5 = strongly agree). Higher scores indicate greater diffusion or stronger perceived performance improvements.

### 3.3 Sampling strategy and data collection

Data were collected from 101 farmers operating in Punjab through a structured questionnaire administered in person and via local agricultural networks. A non-probability convenience sampling approach was adopted because no comprehensive and up-to-date sampling frame of farmers using or considering AI-enabled tools exists in the region, and practical constraints related to the geographic dispersion of farming communities and limited research resources precluded probability-based selection.

Data were collected across Punjab; however, district- and village-level identifiers were not systematically recorded within the final analytical dataset and therefore cannot be reliably reported retrospectively. This limits the geographical specificity of the sample and is acknowledged when interpreting the generalisability of the findings. Convenience sampling is commonly used in exploratory technology-adoption studies in developing agricultural settings where access to respondents is restricted.

The sample included respondents from small, medium and large farming operations, with medium-sized farms representing the largest group, thereby providing variation in farm size and potential technology-adoption conditions.

Although convenience sampling limits statistical generalisability and introduces the possibility of selection bias, several steps were taken to enhance the robustness of the sample. Respondents were drawn from a range of farm sizes and crop types in order to capture variation in adoption behavior and resource endowments. The achieved sample size of 101 exceeds commonly cited minimum thresholds for linear regression analysis involving a small number of predictors (typically 10–15 cases per predictor).

Nevertheless, the relatively modest sample size reduces statistical power for detecting smaller effect sizes and may increase the risk of Type II error. Findings are therefore interpreted as context-specific rather than universally generalisable. Future research employing stratified or probability-based sampling would strengthen external validity.

### 3.4 Instrument design and measurement quality

The questionnaire was developed on the basis of established scales in the digital agriculture and technology adoption literature and was aligned with the study's theoretical framework. Content validity was supported by ensuring that items reflected the core dimensions of AI diffusion, productivity and profitability as conceptualized in the integrated DoI–RBV–TOE model. All items used consistent five-point Likert response formats to facilitate comparability. The individual questionnaire items used to operationalise each construct are provided in Supplementary Table S1.

Internal consistency was assessed using Cronbach's alpha, with a threshold of 0.70 taken as the minimum acceptable level of reliability. Although a formal pilot study was not conducted prior to full data collection, the instrument was carefully reviewed by the research team to minimize ambiguity and ensure clarity of wording for the target respondent group.

To further assess the suitability of the measurement instrument, the Kaiser–Meyer–Olkin (KMO) measure of sampling adequacy and Bartlett's Test of Sphericity were conducted prior to exploratory factor analysis. The KMO value of 0.887 indicated that the data were well suited for factor analysis, while Bartlett's Test of Sphericity was statistically significant (χ^2^ = 665.18, *p* < 0.001), confirming sufficient inter-item correlations among the measurement variables.

The questionnaire items were informed by established literature on innovation diffusion, digital agriculture and farm performance, including Villoria (2019), Benton and Bailey (2019), Araújo et al. (2021), Foster and Rosenzweig (2022), Tzachor et al. (2022), and Karunathilake et al. (2023). The items were subsequently aligned with the dimensions of the integrated DoI–RBV–TOE framework and the three constructs examined in this study.

Future studies should extend the present assessment of measurement validity through exploratory and confirmatory factor analysis using larger and more diverse samples.

### 3.5 Data analysis techniques

Data analysis was conducted using IBM SPSS Statistics and proceeded sequentially in accordance with the study objectives. Descriptive statistics were first used to address the objective of establishing the extent and pattern of AI diffusion and perceived farm performance across the sample. Cronbach's alpha was then used to assess the internal consistency of the multi-item constructs before inferential analysis. Pearson's correlation coefficients were calculated to establish the strength and direction of bivariate associations among AI diffusion, perceived farm productivity and perceived farm profitability. Linear regression was subsequently used to test the three hypothesized associations specified in the conceptual framework.

In line with the conceptual framework, the following regression models were estimated:

**Model 1 (H1):**

Farm Productivity = β0 + β1 (AI Diffusion) + ε

**Model 2 (H2):**

Farm Profitability = β0 + β1 (AI Diffusion) + ε

**Model 3 (H3):**

Farm Profitability = β0 + β1 (Farm Productivity) + ε

AI Diffusion was treated as the independent variable in Models 1 and 2, while Farm Productivity served as the independent variable in Model 3. All constructs were operationalised as composite scores derived from multi-item five-point Likert scales, as detailed in Table 1 (Section 3.2). Given the cross-sectional research design, the correlation and regression analyses permit the identification of statistical associations and the strength and direction of relationships but do not establish causal effects. As each regression model included only a single independent variable, multicollinearity was not a concern. However, additional regression diagnostics, including assessments of residual normality and homoscedasticity, were not undertaken. This limitation is acknowledged, and future research should incorporate more comprehensive diagnostic testing to further strengthen the statistical robustness of the findings.

### 3.6 Ethical considerations

Ethical approval procedures were followed in accordance with standard academic practice. Informed consent was obtained from all participants, who were assured of the voluntary nature of their participation and their right to withdraw at any time. Responses were anonymised and treated confidentially, and data were stored securely. Ethical approval for the study was granted by the ethics committee at Ulster University on 16th February 2026.

## 4 Findings

This section presents the empirical findings of the study. It begins with a summary of the socio-economic characteristics of the respondents, followed by descriptive statistics, reliability assessment, correlation analysis, hypothesis testing and a summary of hypothesis results.

### 4.1 Socio-economic characteristics of the respondents

Table 2 summarizes the socio-economic characteristics of the respondents. The largest age group was 25–34 years (33.7%), followed by 35–44 years (23.8%), indicating that the sample was largely composed of economically active working-age farmers. Most respondents were supervisors (38.6%) or managers (32.7%), while 28.7% identified as farm owners. Medium-sized farms (12–25 acres) represented the largest proportion of the sample (55.4%), followed by large farms (25.7%) and small farms (18.8%). Wheat was the most commonly cultivated crop (34.7%), followed by rice (25.7%), cotton (20.8%) and sugarcane (14.9%). Overall, the sample reflects a diverse cross-section of agricultural operations in Punjab, encompassing variation in managerial responsibility, farm size and cropping activities, thereby providing an appropriate basis for examining AI diffusion and farm performance.

**Table 2:** Respondent characteristics.

Characteristic	Category	*n*	%
Age	18–24	23)	22).8
	25–34	34)	33).7
	35–44	24)	23).8
	45–54	20)	19).8
Role in the farm	Owner	29)	28).7
	Manager	33)	32).7
	Supervisor	39)	38).6
Farm size	Small (< 12 acres)	19)	18).8
	Medium (12–25 acres)	56)	55).4
	Large (>25 acres)	26)	25).7
Main crop	Wheat	35)	34).7
	Rice	26)	25).7
	Cotton	21)	20).8
	Sugarcane	15)	14).9
	Other	4)	4).0

*N* = 101.

### 4.2 Descriptive statistics

Table 3 presents the item-level response distributions for AI diffusion, farm productivity and farm profitability, including frequencies, percentages, means and standard deviations.

**Table 3:** Item-level distribution of farmers' perceptions of AI diffusion, farm productivity and farm profitability.

Construct/questionnaire item	Strongly disagree *n* (%)	D n (%)	N n (%)	A n (%)	SA n (%)	*M*	SD
AI diffusion							
Aware of different AI technologies used in modern agriculture	5 (5.0)	6 (5.9)	37 (36.6)	37 (36.6)	16 (15.8)	**3.52**	**1.00**
Farms use AI tools such as sensors, drones or smart irrigation	8 (7.9)	12 (11.9)	27 (26.7)	41 (40.6)	13 (12.9)	**3.39**	**1.10**
AI improves crop-management decision-making	3 (3.0)	5 (5.0)	21 (20.8)	50 (49.5)	22 (21.8)	**3.82**	**0.93**
AI has increased efficiency in farm operations	3 (3.0)	6 (5.9)	29 (28.7)	36 (35.6)	27 (26.7)	**3.77**	**1.01**
Plan to expand AI use on the farm in future	4 (4.0)	6 (5.9)	27 (26.7)	37 (36.6)	27 (26.7)	**3.76**	**1.04**
Farm productivity							
AI can improve farm productivity	7 (6.9)	8 (7.9)	23 (22.8)	37 (36.6)	26 (25.7)	**3.66**	**1.15**
AI can improve water and fertilizer management	1 (1.0)	6 (5.9)	25 (24.8)	49 (48.5)	20 (19.8)	**3.80**	**0.86**
AI can reduce losses from pests or diseases	1 (1.0)	8 (7.9)	34 (33.7)	35 (34.7)	23 (22.8)	**3.70**	**0.94**
AI can improve farming decisions	1 (1.0)	6 (5.9)	20 (19.8)	48 (47.5)	26 (25.7)	**3.91**	**0.88**
AI can improve monitoring of farm conditions	1 (1.0)	4 (4.0)	20 (19.8)	45 (44.6)	31 (30.7)	**4.00**	**0.87**
Farm profitability							
AI could increase farm profitability	0 (0.0)	7 (6.9)	21 (20.8)	44 (43.6)	29 (28.7)	**3.94**	**0.88**
AI could improve long-term financial sustainability	1 (1.0)	3 (3.0)	20 (19.8)	49 (48.5)	28 (27.7)	**3.99**	**0.83**
AI could improve financial returns	3 (3.0)	4 (4.0)	26 (25.7)	45 (44.6)	23 (22.8)	**3.80**	**0.94**
AI could identify profitable crop-management strategies	1 (1.0)	6 (5.9)	12 (11.9)	59 (58.4)	23 (22.8)	**3.96**	**0.82**
AI could improve overall farm profitability	2 (2.0)	3 (3.0)	22 (21.8)	45 (44.6)	29 (28.7)	**3.95**	**0.90**

*N* = 101. SD, Strongly Disagree; D, Disagree; N, Neutral; A, Agree; SA, Strongly Agree; M, mean; SD (final column), standard deviation. Means and standard deviations were calculated using the five-point Likert scale (1 = Strongly Disagree to 5 = Strongly Agree). Percentages may not total exactly 100% because of rounding.

The item-level findings indicate generally favorable perceptions of AI-enabled agricultural technologies, although reported current use was less widespread than perceived usefulness. For example, 53.5% of respondents agreed or strongly agreed that their farm currently uses AI tools such as sensors, drones or smart irrigation, compared with 71.3% who agreed or strongly agreed that AI improves crop-management decision-making. Similarly, responses concerning farm productivity and profitability were predominantly positive. Agreement was particularly high for AI-enabled monitoring of farm conditions (75.3% agreed or strongly agreed) and for AI's potential to identify profitable crop-management strategies (81.2%). These patterns indicate a distinction between farmers' positive perceptions of AI's potential benefits and the extent to which AI-enabled technologies are currently being used in farming operations.

Table 4 presents the composite descriptive statistics for the three study variables.

**Table 4:** Descriptive statistics.

Variable	*N*	Minimum	Maximum	*M*	SD
AI diffusion	101	7.00	25.00	18.27	3.71
Farm productivity	101	10.00	25.00	19.08	3.66
Farm profitability	101	8.00	25.00	19.64	3.20

N, sample size; M, mean; SD, standard deviation.

AI diffusion recorded a mean composite score of 18.27 (SD = 3.71). The dispersion indicates variation in respondents' reported awareness, use and integration of AI-enabled technologies. Perceived farm productivity recorded a mean composite score of 19.08 (SD = 3.66), while perceived farm profitability recorded the highest mean composite score of 19.64 (SD = 3.20). The dispersion observed across all three constructs indicates variation in farmers' reported experiences of AI diffusion and perceived farm performance, although profitability responses were somewhat more concentrated around the mean.

The individual questionnaire items underlying each composite measure are reported in Supplementary Table S1.

### 4.3 Reliability analysis

Internal consistency was assessed using Cronbach's alpha. As shown in Table 5, all three constructs demonstrated acceptable internal consistency, with Cronbach's alpha coefficients ranging from 0.779 to 0.830. These values exceed the recommended threshold of 0.70, indicating that the measurement scales are reliable for subsequent statistical analyses.

**Table 5:** Reliability analysis.

Construct	Items	Cronbach's α
AI diffusion	5	0.779
Farm productivity	5	0.830
Farm profitability	5	0.784

### 4.4 Correlation analysis

Table 6 presents the Pearson correlation matrix for AI diffusion, farm productivity and farm profitability.

**Table 6:** Pearson correlation matrix.

Variable	1	2	3
1. AI diffusion	1		
2. Farm productivity	0.617^**^	1	
3. Farm profitability	0.607^**^	0.749^**^	1

*N* = 101. ^**^p < 0.001 (2-tailed).

AI diffusion was positively and significantly correlated with farm productivity (*r* = 0.617, *p* < 0.001) and farm profitability (*r* = 0.607, *p* < 0.001). These coefficients indicate moderately strong positive associations, whereby respondents reporting greater AI diffusion also tended to report higher perceived productivity and profitability. Farm productivity demonstrated the strongest association, with farm profitability (*r* = 0.749, *p* < 0.001), indicating that higher perceived productivity was closely associated with higher perceived financial performance.

These findings provide preliminary support for the three hypothesized relationships. However, the correlations represent bivariate associations and do not establish causal relationships. The hypothesized associations are examined further through linear regression analysis in Section 4.5.

### 4.5 Hypothesis testing: linear regression analysis

Linear regression analysis was conducted to examine the hypothesized associations between AI diffusion, farm productivity and farm profitability. Three regression models were estimated in accordance with the conceptual framework. AI Diffusion was specified as the independent variable when predicting Farm Productivity (H1) and Farm Profitability (H2), while Farm Productivity was specified as the independent variable when predicting Farm Profitability (H3). The linear regression results for Model 1 are provided in Table 7.

**Table 7:** Linear regression results for AI diffusion predicting farm productivity.

Predictor	*B*	*t*	*R^2^*	*F*	*p*
AI Diffusion	0.608	7.800	0.381	60.841	< 0.001

*B*, unstandardised regression coefficient; *R*^2^ = coefficient of determination; *F*, F-statistic; *p*, significance level.

#### 4.5.1 Model 1: AI diffusion → farm productivity (H1)

AI Diffusion was a significant positive predictor of Farm Productivity (*B* = 0.608, *t* = 7.800, *p* < 0.001). The model explained 38.1% of the variance in Farm Productivity (*R*^2^ = 0.381; *F* = 60.841, *p* < 0.001), indicating that greater AI diffusion was associated with higher perceived farm productivity. Therefore, H1 is supported. The linear regression results for Model 2 are provided in Table 8.

**Table 8:** Linear regression results for AI diffusion predicting farm profitability.

Predictor	*B*	*t*	*R^2^*	*F*	*p*
AI Diffusion	0.525	7.601	0.368	57.768	< 0.001

B, unstandardised regression coefficient; *R*^2^, coefficient of determination; F, F-statistic; *p*, significance level.

#### 4.5.2 Model 2: AI diffusion → farm profitability (H2)

AI Diffusion was also found to be a significant positive predictor of Farm Profitability (B = 0.525, *t* = 7.601, *p* < 0.001). The regression model explained 36.8% of the variance in Farm Profitability (*R*^2^ = 0.368; *F* = 57.768, *p* < 0.001). These findings indicate that higher levels of AI diffusion are associated with greater perceived farm profitability. Accordingly, H2 is supported. The linear regression results for Model 3 are provided in Table 9.

**Table 9:** Linear regression results for farm productivity predicting farm profitability.

Predictor	*B*	*t*	*R^2^*	*F*	*p*
Farm productivity	0.656	11.247	0.561	126.504	< 0.001

*B*, unstandardised regression coefficient; *R*^2^, coefficient of determination; *F*, F-statistic; *p*, significance level.

#### 4.5.3 Model 3: farm productivity → farm profitability (H3)

Farm Productivity was a significant positive predictor of Farm Profitability (*B* = 0.656, *t* = 11.247, *p* < 0.001). The model explained 56.1% of the variance in Farm Profitability (*R*^2^ = 0.561; *F* = 126.504, *p* < 0.001), demonstrating a strong positive association between improvements in farm productivity and perceived profitability. These findings provide empirical support for H3.

### 4.6 Summary of hypothesis results

Table 10 summarizes the outcomes of the hypothesis testing. The regression analyses provide support for all three proposed hypotheses. AI Diffusion was positively and significantly associated with both Farm Productivity and Farm Profitability, while Farm Productivity was positively and significantly associated with Farm Profitability. Consequently, all three hypotheses were supported at the 0.001 significance level.

**Table 10:** Outcomes of the hypothesis testing.

Hypothesis	Statement	Result
H1	AI diffusion is positively associated with farm productivity.	Supported
H2	AI diffusion is positively associated with farm profitability.	Supported
H3	Farm productivity is positively associated with farm profitability.	Supported

H1, H2, and H3 have been supported by the study findings.

The findings presented in this section provide empirical support for the hypothesized relationships and form the basis for the discussion of their theoretical and practical implications in the following section.

## 5 Discussion

### 5.1 AI diffusion as a structurally constrained process

The findings indicate that AI diffusion in Punjab is neither nascent nor mature, but situated within a transitional phase characterized by uneven and incomplete adoption. While farmers demonstrate relatively high awareness and positive perceptions of AI technologies, actual implementation remains moderate. This divergence reinforces the notion that diffusion is not a purely linear process, as traditionally implied by Diffusion of Innovations (DoI) theory, but is instead mediated by contextual and structural constraints. The observed difference between positive perceptions of AI and comparatively lower reported adoption may reflect contextual barriers identified in previous research, including investment costs, infrastructure limitations, technical capability and institutional support. These factors provide plausible contextual explanations rather than causal mechanisms directly established by the present analysis.

From a DoI perspective, the observed pattern aligns with the early adoption stage, where knowledge and persuasion have progressed, yet decision and implementation stages remain constrained. Unlike the classical DoI assumption that adoption barriers primarily reflect informational or attitudinal limitations, the observed pattern is consistent with previous studies suggesting that structural impediments, including high investment costs, infrastructural deficiencies and institutional gaps, may constrain AI diffusion in developing agricultural settings (Cavazza et al., 2023). These factors were not directly measured in the present study but provide a plausible contextual explanation. In contrast to developed contexts, where diffusion is often driven by market incentives and competitive pressures, the observed pattern is consistent with previous literature suggesting that adoption in Punjab may be influenced by external structural constraints.

The concept of “constrained diffusion” therefore emerges as a more appropriate analytical lens. Diffusion is conditional upon enabling environments rather than simply driven by innovation attributes. This reframing shifts the focus of analysis and policy from increasing awareness alone to removing structural bottlenecks that limit implementation.

### 5.2 AI diffusion and farm productivity

The regression analysis demonstrated a statistically significant positive association between AI diffusion and farm productivity, supporting H1. AI diffusion explained 38.1% of the variation in perceived farm productivity (*R*^2^ = 0.381, *p* < 0.001), suggesting that greater integration of AI-enabled agricultural technologies is associated with improved operational performance. These findings are consistent with previous studies demonstrating that AI-driven practices, including precision irrigation, predictive analytics and sensor-based monitoring, can improve input-use efficiency, resource allocation and production management (Araújo et al., 2021; Karunathilake et al., 2023).

From a Resource-Based View (RBV) perspective, these productivity gains are not solely a function of technological adoption but are contingent upon the presence of complementary capabilities. AI technologies enhance productivity by augmenting existing farm resources, including land, labor and production inputs, rather than replacing them. This reinforces the view of AI as an enabling rather than a substitutive innovation. At the same time, the reliance on perceptual measures introduces the possibility of optimism bias, particularly in early stages of adoption where expectations may exceed realized outcomes. This interpretation is consistent with the literature on technological learning curves, which emphasizes the role of experimentation and capability accumulation in realizing productivity gains (Tzachor et al., 2022).

Variation in perceived productivity scores may reflect differences in farm resources and organizational capabilities, as suggested by the RBV literature. However, these factors were not directly measured in the present study.

### 5.3 AI diffusion and farm profitability

The regression results demonstrated a statistically significant positive association between AI diffusion and farm profitability, supporting H2. AI diffusion explained 36.8% of the variation in perceived farm profitability (*R*^2^ = 0.368, *p* < 0.001). These findings extend the existing literature, which has largely concentrated on productivity outcomes, by indicating that AI diffusion is also associated with improved perceived financial performance. The observed relationship is consistent with previous studies suggesting that AI-enabled technologies may enhance profitability through more efficient resource utilization, reduced input waste and improved operational decision-making rather than through output expansion alone (Benton and Bailey, 2019).

The relationship between AI diffusion and profitability should be interpreted with appropriate caution. Previous research suggests that factors such as high implementation costs, ongoing maintenance requirements and the availability of technical expertise may influence the extent to which productivity improvements translate into financial returns. These factors were not directly measured in the present study but provide plausible contextual explanations for variation in profitability outcomes reported elsewhere in the literature. Similarly, RBV proposes that value creation depends not only on access to technology but also on complementary organizational resources and capabilities, including financial, human and infrastructural assets. Consequently, while the present findings demonstrate a positive association between AI diffusion and profitability, future research should examine the contextual conditions under which these financial benefits are realized and whether they differ across farm types and resource endowments.

### 5.4 The interdependence of productivity and profitability

The strongest relationship observed in this study was between farm productivity and farm profitability. The regression analysis showed that farm productivity explained 56.1% of the variation in farm profitability (*R*^2^ = 0.561, *p* < 0.001), supporting H3. This finding is consistent with previous research demonstrating that productivity and profitability are closely related but analytically distinct dimensions of farm performance, with the translation of productivity gains into financial returns dependent on production costs, relative prices and broader market conditions (Mugera et al., 2016; Foster and Rosenzweig, 2022). This finding suggests that productivity and profitability are closely associated dimensions of farm performance, with improvements in operational efficiency corresponding to improved financial outcomes. The strength of this relationship indicates that productivity may represent an important pathway through which AI diffusion is associated with profitability.

The findings also suggest that farms reporting higher levels of productivity tend to report higher levels of profitability. Previous studies have proposed that improved financial performance may facilitate continued investment in AI technologies and other innovation capabilities, potentially reinforcing competitive advantage over time. Conversely, the literature indicates that financial, infrastructural and technical constraints may limit the ability of some farms to sustain technology adoption and realize comparable benefits. As these contextual factors were not directly examined in the present study, they should be regarded as literature-informed interpretations rather than empirical findings. Collectively, these observations are consistent with insights from DoI, RBV and dynamic capability perspectives, which conceptualize AI adoption as an evolving process of capability development and organizational learning. Conversely, given the cross-sectional nature of the data, these potential dynamics should be interpreted cautiously and warrant examination through longitudinal research.

### 5.5 Structural barriers and the diffusion–performance gap

One of the most significant contributions of this study is the identification of a diffusion–performance gap, whereby farmers reported favorable perceptions of AI technologies while levels of AI diffusion remained comparatively moderate. This pattern suggests that positive perceptions alone may not be sufficient to translate into widespread adoption or sustained improvements in farm performance. From a Technology–Organization–Environment (TOE) perspective, this observed gap is consistent with the proposition that environmental and institutional conditions influence technology adoption alongside organizational and technological factors.

Previous studies have identified high capital investment requirements, limited digital infrastructure and connectivity, unreliable electricity supply, and weak agricultural extension systems as important constraints affecting AI adoption in developing agricultural settings (Tzachor et al., 2022; Gardezi et al., 2024). Although these factors were not directly measured in the present study, they provide plausible contextual explanations for the diffusion–performance gap observed in Punjab. The findings support broader calls for a systems-based approach to digital agriculture in which technological innovation is accompanied by appropriate infrastructure, institutional support and knowledge dissemination mechanisms. Such an approach may facilitate more effective and sustained AI diffusion rather than simply increasing awareness or initial adoption.

Accordingly, improving access to digital agricultural technologies requires attention not only to technology availability but also to the conditions that enable farmers to use such technologies effectively. The literature indicates that improved rural connectivity, reliable energy infrastructure, affordable financing, strengthened agricultural extension services, digital capability development and shared-access models may reduce barriers to participation, particularly for resource-constrained farmers. These access mechanisms are considered further in the policy and practical implications presented in Section 6.

From the perspective of Diffusion of Innovations (DoI) theory, the observed relationships are consistent with the proposition that perceived relative advantage, compatibility and complexity influence technology adoption decisions. Likewise, the Resource-Based View (RBV) suggests that the benefits derived from AI technologies are likely to depend upon complementary organizational capabilities and resource availability. While these theoretical perspectives provide useful explanations for the patterns observed in this study, the specific technological, organizational and environmental factors underpinning AI diffusion were not directly examined. Future research should therefore investigate these constructs explicitly to better understand the mechanisms through which AI adoption influences farm performance across different agricultural contexts.

## 6 Conclusion and recommendations

### 6.1 Conclusion

This study examined the diffusion of artificial intelligence (AI)-enabled agricultural technologies among farmers in Punjab, Pakistan, and assessed the associations between AI diffusion, perceived farm productivity and perceived farm profitability. Drawing on an integrated framework combining Diffusion of Innovations (DoI), the Resource-Based View (RBV) and the Technology–Organization–Environment (TOE) framework, the study provides context-specific evidence from a developing agricultural economy in which the diffusion of AI-enabled technologies remains uneven.

The empirical findings demonstrate statistically significant positive associations between AI diffusion and both perceived farm productivity and perceived farm profitability. AI diffusion explained 38.1% of the variation in perceived farm productivity and 36.8% of the variation in perceived farm profitability. Farm productivity was also strongly and positively associated with farm profitability, explaining 56.1% of its variation. These findings support all three hypotheses and indicate that respondents reporting greater AI diffusion also tended to report stronger perceived operational and financial performance.

The descriptive findings further reveal an important distinction between farmers' perceptions of AI and its reported use. Respondents generally expressed favorable views regarding the potential contribution of AI-enabled technologies to farm productivity and profitability, while reported current use was comparatively lower. This perception–adoption gap is consistent with previous research identifying financial, infrastructural, technical and institutional constraints to digital agricultural adoption. However, because these contextual barriers were not directly measured in the present study, they should be interpreted as literature-informed explanations rather than empirically established determinants of adoption within the surveyed sample.

The findings also reinforce the value of examining productivity and profitability as related but distinct dimensions of farm performance. The strong association between the two suggests that perceived improvements in operational performance are closely related to perceived financial outcomes, while the integrated DoI–RBV–TOE framework provides a useful basis for interpreting AI diffusion within its broader organizational and institutional context.

These conclusions should be interpreted within the methodological boundaries of the study. The cross-sectional design identifies statistical associations rather than causal effects, the outcome measures are perceptual rather than objective measures of farm performance, and the non-probability convenience sample of 101 respondents limits statistical generalisability beyond the surveyed population. Future research employing longitudinal designs, objective performance measures and probability-based sampling across multiple agricultural regions would strengthen causal inference and external validity.

### 6.2 Policy implications

When considered alongside the structural constraints identified in the existing literature, the study findings point to several priority areas for policy attention. Reliable broadband connectivity and electricity supply are foundational prerequisites for meaningful AI use; without them, even well-designed tools remain inaccessible to many farmers. Targeted financial instruments such as subsidies, tax incentives, or low-interest credit lines are needed to lower the high upfront costs that currently exclude smaller operators. In parallel, agricultural extension systems should be strengthened and reoriented toward practical digital skills training so that the gap between awareness and technical capability can be reduced. Finally, policy support for public–private partnerships can encourage the development of affordable, context-specific AI solutions suited to the predominance of smallholder farming in Punjab, including applications that address climate-related risks such as water scarcity and yield variability.

### 6.3 Practical implications

For farmers, the positive associations identified in this study, considered alongside the wider literature on AI-enabled agriculture, suggest that selective and incremental adoption of AI tools, particularly those that deliver visible efficiency gains in irrigation, pest management, or input use, can contribute to improved productivity and cost control. Given the importance of observability and trialability in diffusion processes, technologies that produce clear, demonstrable results are more likely to gain traction. At the same time, farmers should anticipate a learning period during which productivity may temporarily dip as new skills and routines are developed. Collective arrangements, such as cooperative access to equipment or shared digital advisory services, may help lower individual cost and capability barriers.

### 6.4 Recommendations

Drawing on the empirical findings together with the contextual barriers identified in the literature, several practical steps are recommended to support more inclusive and effective AI diffusion. First, investment in rural digital infrastructure and reliable energy supply should be prioritized. Second, financial mechanisms tailored to small and medium-sized farms are required to address capital constraints. Third, structured training programmes and expanded digital extension services are essential to build the human capabilities needed for effective use. Fourth, shared-access or AI-as-a-service models, together with low-cost modular technologies, can help broaden participation beyond better-resourced commercial farms. Finally, stronger collaboration between government, research institutions, and agritech providers is needed to ensure that solutions are both technically sound and contextually appropriate.

Future research should address the methodological limitations of the present study by employing longitudinal designs, incorporating objective performance measures alongside perceptual data, applying more advanced analytical techniques capable of addressing endogeneity, and examining moderating factors such as farm size, education, and infrastructure access. Such work would strengthen causal inference and improve the generalisability of findings on AI diffusion in developing agricultural systems.

## Data Availability

The raw data supporting the conclusions of this article will be made available by the authors, without undue reservation.
