# Supplementary material for: Improving farm productivity and profitability through AI diffusion: evidence from Punjab, Pakistan
Source: Front Artif Intell. 2026 Oct 21;9:1890862. doi: 10.3389/frai.2026.1890862 (PMC13731396; doi:10.3389/frai.2026.1890862)
Supplement: Supplementary file 1 [file Data_Sheet_1.docx]

**Appendix A**

**Supplementary Table S1**. *Survey Items and Construct Measurement*

| **Construct** | **Survey item** | **Response scale** |
| --- | --- | --- |
| **AI Diffusion** | I am aware of different AI technologies used in modern agriculture. | 1 = Strongly Disagree to 5 = Strongly Agree |
|  | My farm uses AI tools such as sensors, drones or smart irrigation. | 1 = Strongly Disagree to 5 = Strongly Agree |
|  | AI improves crop-management decision-making. | 1 = Strongly Disagree to 5 = Strongly Agree |
|  | AI has increased efficiency in farm operations. | 1 = Strongly Disagree to 5 = Strongly Agree |
|  | I plan to expand AI use on the farm in the future. | 1 = Strongly Disagree to 5 = Strongly Agree |
| **Farm Productivity** | AI can improve farm productivity. | 1 = Strongly Disagree to 5 = Strongly Agree |
|  | AI can improve water and fertiliser management. | 1 = Strongly Disagree to 5 = Strongly Agree |
|  | AI can reduce losses from pests or diseases. | 1 = Strongly Disagree to 5 = Strongly Agree |
|  | AI can improve farming decisions. | 1 = Strongly Disagree to 5 = Strongly Agree |
|  | AI can improve monitoring of farm conditions. | 1 = Strongly Disagree to 5 = Strongly Agree |
| **Farm Profitability** | AI could increase farm profitability. | 1 = Strongly Disagree to 5 = Strongly Agree |
|  | AI could improve long-term financial sustainability. | 1 = Strongly Disagree to 5 = Strongly Agree |
|  | AI could improve financial returns. | 1 = Strongly Disagree to 5 = Strongly Agree |
|  | AI could identify profitable crop-management strategies. | 1 = Strongly Disagree to 5 = Strongly Agree |
|  | AI could improve overall farm profitability. | 1 = Strongly Disagree to 5 = Strongly Agree |

**Note.** All items were measured using a five-point Likert scale ranging from 1 (Strongly Disagree) to 5 (Strongly Agree). Five items were used to operationalise each construct. Composite scores were calculated across the five constituent items, with higher scores indicating greater AI diffusion or stronger perceived farm productivity and profitability.
